# Can Pediatric Surgeons Become Truly Experienced for Thyroid Surgery on a Universal Scale?

**DOI:** 10.4274/jcrpe.5381

**Published:** 2018-05-18

**Authors:** İrem İnanç, Atakan Sezer, Mustafa İnan

**Affiliations:** 1 Trakya University Faculty of Medicine, Department of Pediatric Surgery, Edirne, Turkey; 2 Trakya University Faculty of Medicine, Department of General Surgery, Edirne, Turkey

**Keywords:** Thyroid nodule, thyroidectomy, surgical experience, pediatric surgeon

**To the Editor,**

We have read with great interest the paper titled “Management of Childhood Thyroid Nodules: Surgical and Endocrinological Findings in a Large Group of Cases” (1). This paper retrospectively analysed 103 children with thyroid nodules admitted to the authors’ centre over a period of 9 years and indicated that there would be fewer resulting complications in more experienced hands. We have evaluated, from our perspective, the type of “experience” described in the paper as important for the success of pediatric thyroid surgeries.

Careful examination of the literature clearly shows the most significant criterion influencing experience in thyroid surgery is the number of patients operated on annually. Further, it is indicated that the fundamental criteria of surgical success are low rates of recurrent laryngeal nerve injury, post-operative hypocalcaemia and hospitalization time (2); the rate at which complications occur is inversely proportional to experience.

Surgeons interested in thyroid surgery have been categorized by researchers as belonging to different groups, such as high-volume surgeons, pediatric surgeons and others (3). For example, endocrine surgeons are a group found to perform an average of more than 100 thyroid surgeries per year (3,4). Tuggle et al (3) indicated that high-volume surgeons perform at least 30 thyroid surgeries and an average of 2 pediatric endocrine surgeries per year. Research has found that pediatric surgeons perform, on average, 2 pediatric thyroid surgeries per year. The same study emphasized that there were no pediatric surgeons in the high-volume group (3). 

In conclusion, we think that it is not possible to categorize pediatric surgeons in the “experienced surgeon” or “endocrine surgeon” groups for thyroid surgery because pediatric surgeons do not have enough patient potential to complete a high volume of thyroid surgeries annually, as defined by the international literature. If thyroid surgeries are performed by pediatric surgeons without assistance, we believe that pediatric patients will frequently encounter recurrent laryngeal nerve injury, post-operative hypocalcaemia and long-term hospitalization issues. While experience is the most significant criteria in reducing the rate of complications in thyroid surgery, it is evident that a multidisciplinary approach is necessary for pediatric patients with different physiologies than adults. For optimal results, therefore, surgery on such patients, particularly for total thyroidectomy and lymph node and neck dissection operations, should be conducted by a team comprising pediatric surgeons and high-volume thyroid or endocrine surgeons.
